# Association between sensory function and medio-lateral knee position during functional tasks in patients with anterior cruciate ligament injury

**DOI:** 10.1186/1471-2474-15-430

**Published:** 2014-12-13

**Authors:** Anna Cronström, Eva Ageberg

**Affiliations:** Department of Health Sciences, Lund University, PO Box 157, Lund, SE-221 00 Sweden

**Keywords:** Proprioception, Sensory function, Kinematics, Anterior cruciate ligament, Movement quality

## Abstract

**Background:**

Patients with anterior cruciate ligament (ACL) injury often exhibit reduced movement quality during functional tasks in the form of a knee-medial-to-foot position (KMFP). This movement pattern is suggested to be more common in women than in men, but the possible contributing sensorimotor factors for this altered knee position are poorly studied in these patients. The aim of this study was to evaluate the association between sensory function and medio-lateral knee position during functional tasks in men and women with ACL injury.

**Methods:**

Fifty-one patients (23 women) aged 18–40 years with ACL injury were included in this cross-sectional study. Measures of sensory function were assessed by the threshold to detection of passive motion (TDPM) for knee kinesthesia and by the vibration perception threshold (VPT) for vibration sense. Movement quality was assessed by visual observation of the position of the knee relative to the foot during the following four functional tasks with different degrees of difficulty: the single-limb mini-squat, stair descending, the forward lunge, and the drop-jump. Spearman’s rank correlation coefficient was used to determine the relationship between the sensory measures and the medio-lateral knee position during the functional tasks. Differences in TDPM and/or VPT between subjects with good and poor movement quality were evaluated using the independent *t*-test. Separate gender analyses were performed.

**Results:**

Worse TDPM was associated with a KMFP during the drop jump in men. Worse VPT at the toe and ankle was associated with a KMFP during stair descending and the forward lunge in women, but no associations were found in men.

**Conclusion:**

Worse kinesthesia, measured by TDPM, might be associated with KMFP during the drop jump in men with ACL injury while worse vibration sense, measured by the VPT, at the foot and ankle might be related to KMFP in women. Further studies are needed to confirm these results.

**Electronic supplementary material:**

The online version of this article (doi:10.1186/1471-2474-15-430) contains supplementary material, which is available to authorized users.

## Background

It is well known that patients with ACL injury have decreased functional ability such as reduced muscle strength or a shorter hop distance with the injured leg compared to the non-injured leg as assessed by quantitative methods [1]. Moreover, one third of these patients will never get back to their pre-injury activity level [2]. Recent investigations report that these patients also exhibit poorer quality of movement during functional tasks as measured by changes in postural orientation [3–5]. Postural orientation is defined as the “ability to stabilize the body segments in relation to each other and the environment”, for instance, keeping the trunk, hip, knee, and ankle in line during dynamic activity [6]. The medio-lateral knee position is commonly used to evaluate movement quality of the knee [7–9], and a knee-over-foot position (KOFP) reflects good movement quality while a knee-medial-to-foot position (KMFP) reflects poor movement quality. A KMFP during movements has been reported to be a risk factor for sustaining an ACL injury [9, 10] and to be related to a high re-injury rate [11, 12]. Three-dimensional (3-D) motion analysis equipment is the gold standard for measuring medio-lateral knee position, but two-dimensional (2-D) motion analysis and visual observation and scoring are also used. The latter has moderate to high reliability [4, 7, 8, 13, 14], is valid in 2-D [7], and is an inexpensive method that is easy to use in both clinical settings and in large-scale studies [4, 7].

Possible contributing factors for medio-lateral knee position in patients with ACL injury are not well studied, but such data would be helpful in the design of training regimes. In a previous study, no relation was found between quadriceps or hamstring muscle power and postural orientation during several different functional activities [3], and this indicates that factors other than muscle power contribute to the altered movement quality in these patients.

Sensory function in terms of knee proprioception is impaired after ACL injury [15]. Kinesthesia, measured as the threshold to detection of passive motion (TDPM) is commonly used to assess proprioception in patients with an ACL-deficient knee [16, 17]. In a recent study, sense of vibration was evaluated as a measure of sensory function in patients with an ACL injury [18]. It was reported that worse proprioception is related to worse functional performance—as measured by the one-leg hop test for distance [19, 20]—and to poorer single-limb balance [21] in patients with ACL injury. Hence, proprioceptive acuity seems to be relevant for functional performance. Moreover, a previous study reported a relation between worse proprioception and reduced ability to control knee flexion during a single-leg stop jump task, in non-injured men, [22]. However, to our knowledge, the possible relation between sensory function and medio-lateral knee position during functional tasks has not been previously investigated. Because previous studies have found that a KMFP was more prevalent in women than in men during activity in both non-injured individuals [23–27] and in patients with ACL injury [5], it is important to analyze men and women separately.

The aim of this cross-sectional study was to investigate the association between sensory function—measured as kinesthesia and vibration sense—and medio-lateral knee position during functional tasks in patients with ACL injury. Possible gender differences were also explored. We hypothesized that worse sensory function would be related to a KMFP during functional tasks and that this relation would be more evident in women than in men.

## Methods

### Subjects

A convenience sample of 51 patients (23 women) ranging in age from 18 years to 40 years with arthroscopic or MRI-verified ACL injury or ACL reconstruction with or without associated injuries to the knee and currently undergoing neuromuscular training, supervised by physical therapists, were included (Table 1). Patients who used crutches, those who had finished their rehabilitation, and those with other injuries affecting function were excluded. Men were approximately 3 years older and had a higher BMI than women, but no other demographic differences were present (Table 1). All patients were recruited at sports physical therapy clinics in Skåne, Sweden, and gave their written informed consent to participate. The study was approved by the Advisory Committee for Research Ethics in Health Education at the Faculty of Medicine of Lund University (VEN 48–12).

**Table 1:** **Characteristics of the subjects**

Characteristic	Women (n = 23)	Men (n = 28)	p-value
Age (years)¤	23 (4.0)	26 (6.1)	**0.024**
BMI¤	22.5 (2.21)	24.7 (2.85)	**0.004**
Tegner activity score pre injury *#*	9 (8–9)	9 (8–9)	0.504
Tegner activity score post injury *#*	3 (2–6)	4 (3–7)	0.274
Injured right knee, *n (%)*	11 (48)	20 (71)	0.089
Time since injury, non-reconstruction (weeks)¤	45 (42.1)	66 (57.9)	0.146
Reconstruction, *n (%)*	19 (83)	19 (68)	0.234
Time since reconstruction (weeks)¤	36 (40.7)	47 (52.5)	0.474
Associated injuries, *n (%)*	13 (57)	20 (71)	0.272
*Collateral ligament, n (%)*	5 (22)	8 (28.6)	0.581
*Meniscal, n (%)*	9 (39)	16 (57.1)	0.160
*Cartilage, n (%)*	8 (35)	7 (25)	0.450

¤ = mean (SD), # = median (quartiles), bold text = statistically significant difference (p ≤ 0.05).

### Assessment

The measurements were taken in the order that they appear below. A 5-minute warm up on a stationary bike preceded the evaluation of the functional tasks. All subjects wore shorts and performed the single leg squat and stair descending barefoot. For the forward lunge and drop jump, the participants wore their training shoes for shock absorption. The sensory measures were taken by one investigator who was blinded to the movement quality scores. A consensus assessment was reached by two raters who were blinded to the sensory scores, and this was used to determine the medio-lateral knee position scores. Data for the injured leg were used in the analysis. The images in this study illustrate the methods used. These individuals were not included in the data collection. All gave their written consent to publish these images.

### Vibration sense

The vibratory perception threshold (VPT) was measured with a biothesiometer [28–31] according to the manufacturer’s instructions. The application button on the biothesiometer was held in such way that the weight of the machine provided the application button with a standard pressure. To make the subject familiar with the biothesiometer, it was tested on the subjects’ processus styloideus ulna prior to the trials. The vibrator was then held to the subjects’ most prominent point of the metatarsophalangeal joint 1 (MTP1), the medial malleolus (MM), and the medial femoral condyle (MF). The subjects lay in a supine position with their eyes closed and were told to indicate by raising their hand when any sensation of vibration was felt [31]. The amplitude was increased by 1 volt per second, and the voltage when the subject first felt any sensation of vibration was noted as the VPT. The mean of three subsequent measurements was calculated for statistics analysis. A higher value indicated worse VPT. High reliability (ICC = 0.96–0.99) for the biothesiometer has been reported [32].

### Kinesthesia

**Figure 1:**
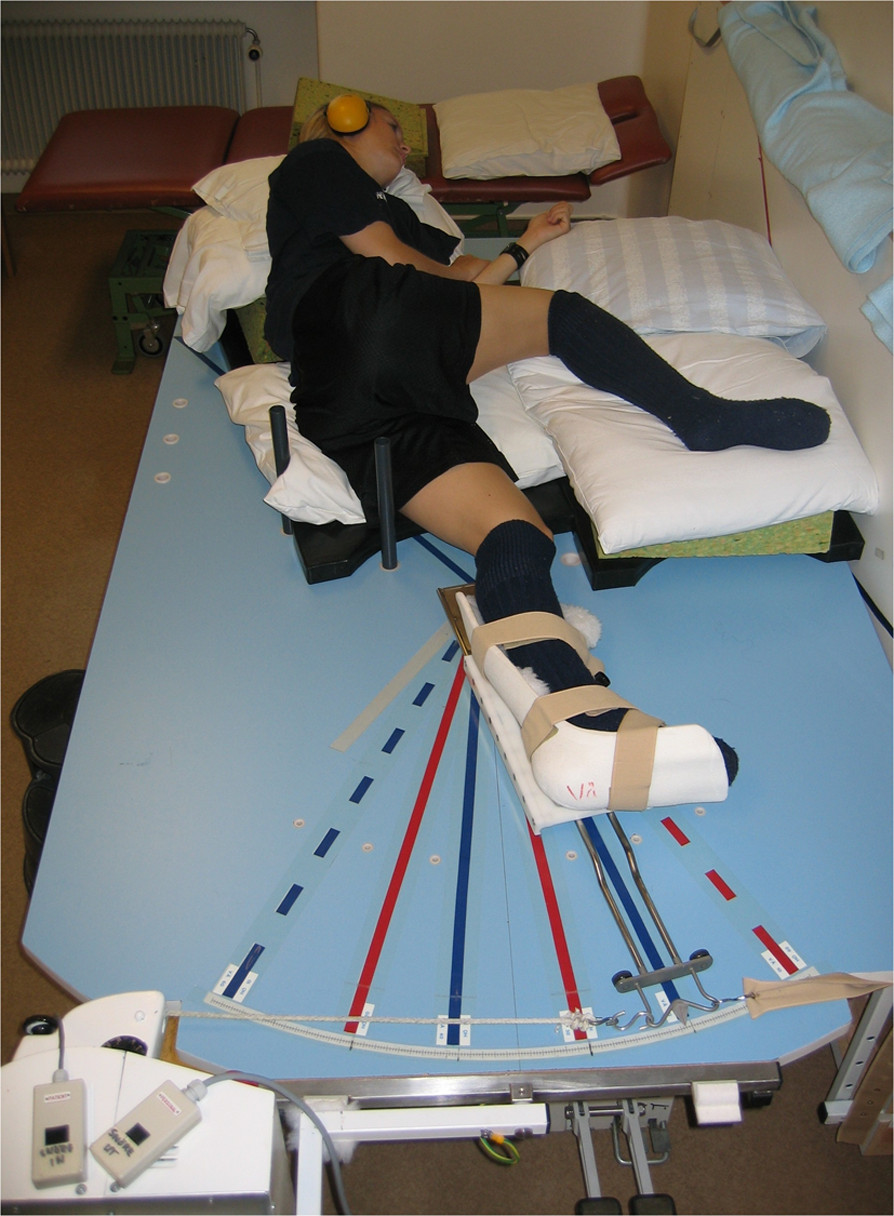
**Kinesthesia testing device.**

Kinesthesia was measured by the TDPM on a specially designed platform as described previously [16] (Figure 1). The platform is mounted on a steel frame and has an electric motor with a wire mounted at the end. The subject lies on the platform in a lateral position with the lower leg in a plastic splint, and the splint is attached to a sled and connected to the wire that can make the splint move the knee in either flexion or extension. An analog scale at the end of the platform registers movements in increments of 0.25°. In order to prevent any false experience of movements derived from the sound when the measuring device was started, all participants wore headphones and listened to a recording of the sound that was produced by the electric motor. The subjects were told to close their eyes and indicate by raising their hand when any movement in the knee was felt. TDPM was measured towards extension (TE) and flexion (TF) from a 20-degree starting position [16]. The median values of three consecutive measurements of TE20 and TF20 were determined, and an index value created from the sum of TE20 and TF20 was used for the statistical analysis. A higher value indicates poorer TDPM, and moderate reliability (ICC = 0.63–0.70) has previously been reported for this device [16].

### Movement quality

**Figure 2:**
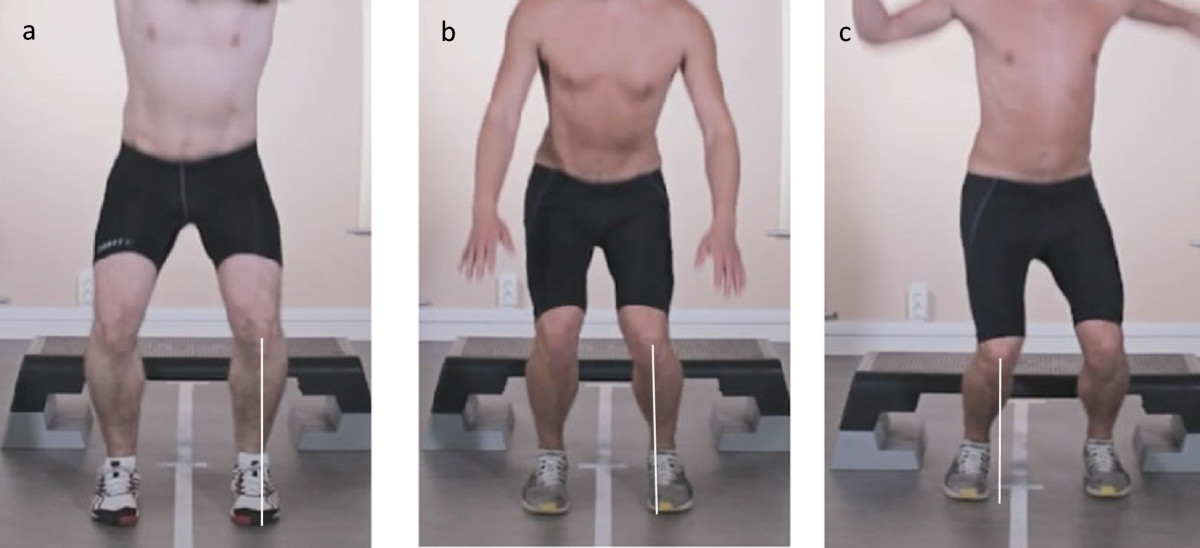
**The position of the knee in relation to the foot during the drop jump. a)** A score of 0, representing good movement quality. **b)** A score of 1, representing fair movement quality. **c)** A score of 2, representing poor movement quality.

Movement quality, in terms of postural orientation, was assessed by visual observation of a video recording of each trial. The raters were able to watch the video in slow motion and to review the movie as many times as needed. The position of the knee in relation to the foot was independently scored by two experienced assessors during four different functional tasks commonly used to assess medio-lateral knee position. The tasks included the single-limb mini squat [7], stair descending [33], the forward lunge [34], and the drop-jump [9] (Table 2). The knee position relative to the foot during the participant’s performance of each task was assessed on an ordinal scale from 0 to 2. If the mid-point of the patella was in line with or lateral to the second toe, a score of 0 = “good” was given for the movement quality. If the mid-point of the patella was medial to the second toe, a score of 1 = “fair” was given. Finally, if the mid-point of the patella was clearly placed medial to the first toe, a score of 2 = “poor” was given (Figure 2). Any disagreement was resolved by consensus discussion between the two raters. If required, the recording was viewed several times and/or in slow motion until consensus was reach. These movement quality assessments showed good to excellent agreement (ICC = 0.710–0.939) (Table 3).

**Table 2:** **Description of the functional tasks**

Single limb mini squat	Performed according to Ageberg et al. [7] but without allowing fingertip support for maintaining balance. The patient is standing on one leg with the second toe placed on a marked longitudinal line. He/she is instructed to flex the injured knee until he/she cannot see the toes (approximately 50°) and then to return to extension. Repeated 5 times.
Stair descending	Performed according to Pfeifer et al. [33] and modified to use a step board 27 cm off the ground. The patient stands on the step board. He/she is instructed to step down to the floor with the non-injured leg and then return to the starting position. The injured leg, which is in contact with the step board throughout the entire movement, is evaluated. Repeated 5 times.
Forward lunge	Performed according to Alkjaer et al. [34]. The patient is standing with his/her feet hip-width apart on the floor. He/she is instructed to take a long stride forward, about 1 m, with the injured leg and to flex the knee to approximately 90° and then push back to the starting position by extending the front leg. Repeated 3 times.
Drop jump	Performed according to Hewett et al. [9] but modified to use the second landing instead of the first landing, because the second landing may better represent the situation when an ACL injury occur [35]. The patient stands on a step board 27 cm off the ground with the feet hip-width apart. He/she is instructed to drop from the step board and directly perform a maximal vertical jump. Arm swing is allowed during the jump. Repeated 3 times.

**Table 3:** **Intraclass correlation coefficient and 95**% **confidence interval for inter-rater reliability for the knee position score assessment**

Test	ICC (95%CI)
	Knee assessment of injured leg
Single-limb mini squat	0.757 (0.572-0.861)
Stair descending	0.780 (0.616-0.874)
Forward lunge	0.716 (0.278-0.868)
Drop jump	0.939 (0.885-0.967)

For safety reasons, subjects who had an ACL reconstruction less than six months prior to the test occasion or those whose physical therapist stated that they were not ready to jump were not permitted to perform the drop jump test. Subjects who were not able to perform the test were considered as missing values.

### Statistics

All calculations were performed with SPSS statistics 20.0. There were no statistically significant correlations between patient demographics and the medio-lateral knee position or sensory variables (p > 0.05). Men and women were analyzed separately. Spearman’s rank correlation coefficient was used to determine the relationship between the sensory measures and the medio-lateral knee position during the functional tasks. The knee position scores were also dichotomized for each test; one group included subjects who were given a score of “0” (good movement quality), and one group included subjects who had been given a score of either “1” or “2” (poor movement quality). These values were used to evaluate any differences in TDPM and/or VPT between subjects with good and poor movement quality using the independent *t*-test. The independent *t*-test was also used to evaluate any gender difference in sensory function. The Mann–Whitney *U*-test was used to detect any differences in medio-lateral knee position between men and women. Because this was an exploratory study, we did not calculate the sample size a priori, nor did we apply corrections for multiple comparisons. A p-value less than or equal to 0.05 was considered statistically significant.

## Results

All 51 subjects completed the tests of sensory function. Sixteen subjects were not able to perform all functional tasks, especially the forward lunge and the drop jump. There was no gender difference in medio-lateral knee position for any of the functional tasks (Table 4).

**Table 4:** **Sensory function and knee position score data during the functional tasks for women and men**

	Women (n = 23)	Men (n = 28)	
Sensory test (n = 51)	Mean (SD)		p-value
TDPM (degrees)	2.43 (1.38)	1.91 (0.87)	0.105
VPT-MTP 1 (volts)	5.04 (2.33)	5.27 (2.88)	0.754
VPT-MM (volts)	7.14 (2.57)	8.77 (4.41)	0.124
VPT-MF (volts)	12.17 (3.66)	14.0 (4.05)	0.098
**Functional task**	**Median (quartiles, range)**		
Single-limb mini squat	1 (0–1, 0–2) (n = 23)	1 (0–2, 0–2) (n = 28)	0.116
Stair descending	0 (0–1, 0–1) (n = 22)	0 (0–1, 0–2) (n = 28)	0.675
Forward lunge	1 (0–1, 0–1) (n = 19)	1 (0–1, 0–2) (n = 28)	0.824
Drop jump	0 (0–1, 0–2) (n = 13)	1 (0–1, 0–2) (n = 23)	0.662

TDPM = threshold to detection of passive motion, VPT = vibration perception threshold, MTP1 = metatarsophalangeal joint 1, MM = medial malleolus, MF = medial femoral condyle, bold text = statistically significant difference (p ≤ 0.05).

### Correlations between sensory function and movement quality

Worse kinesthesia was associated with a KMFP during the drop jump in men (r_s_ = 0.423, p = 0.044) (Table 5). In women, worse vibration sense at MTP1 and MM was associated with a KMFP during stair descending (r_s_ = 0.453, p = 0.034 for MTP1, and r_s_ = 0.626, p = 0.002 for MM), and worse vibration sense at MM was associated with a KMFP during the forward lunge (r_s_ = 0.544, p = 0.016) (Table 5).

**Table 5:** **Correlations between sensory function and medio-lateral knee position during the functional tasks**

	Medio-lateral knee position			
Sensory test				
	Single-limb mini squat	Stair descending	Forward lunge	Drop jump
***Women***	*(n = 23)*	*(n = 22)*	*(n = 19)*	*(n = 13)*
**TDPM (degrees)**	r_s_ = 0.344	r_s_ = −0.288	r_s_ = 0.000	r_s_ = 0.469
	p = 0.108	p = 0.194	p = 1.0	p = 0.106
**VPT MTP1 (volts)**	r_s_ = 0.114	**r**_**s**_ **= 0.453**	r_s_ = 0.308	r_s_ = −0.343
	p = 0.603	**p = 0.034**	p = 0.199	p = 0.251
**VPT MM (volts)**	r_s_ = 0.069	**r**_**s**_ **= 0.626**	**r**_**s**_ **= 0.544**	r_s_ = −0.088
	p = 0.756	**p = 0.002**	**p = 0.016**	p = 0.776
**VPT MF (volts)**	r_s_ = −0.092	r_s_ = 0.313	r_s_ = 0.409	r_s_ = 0.408
	p = 0.675	p = 0.155	p = 0.082	p = 0.167
***Men***	*(n = 28)*	*(n = 28)*	*(n = 28)*	*(n = 23)*
**TDPM (degrees)**	r_s_ = 0.008	r_s_ = 0.338	r_s_ = −0.030	**r**_**s**_ **= 0.423**
	p = 0.969	p = 0.079	p = 0.878	**p = 0.044**
**VPT MTP1 (volts)**	r_s_ = −0.170	r_s_ = −0.143	r_s_ = −0.052	r_s_ = −0.069
	p = 0.388	p = 0.468	p = 0.794	p = 0.755
**VPT MM (volts)**	r_s_ = −0.115	r_s_ = 0.021	r_s_ = 0. − 232	r_s_ = −0.162
	p = 0.560	p = 0.917	p = 0.235	p = 0.461
**VPT MF (volts)**	r_s_ = −0.039	r_s_ = 0.121	r_s_ = −0.237	r_s_ = 0.338
	p = 0.844	p = 0.540	p = 0.224	p = 0.079

TDPM = threshold to detection of passive motion, VPT = vibration perception threshold, MTP1 = metatarsophalangeal joint 1, MM = medial malleolus, MF = medial femoral condyle, r_s_ = Spearman’s rank correlation coefficient, bold text = statistically significant difference (p ≤ 0.05).

### Differences in sensory function between subjects with good and poor movement quality

**Figure 3:**
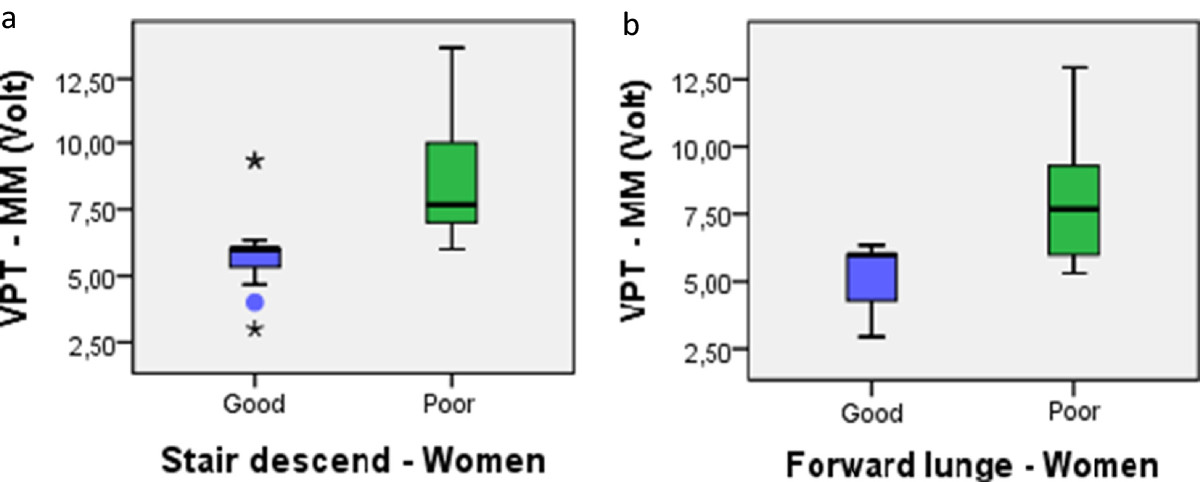
**Differences in vibration sense between women with good and poor movement quality during stair descending (a) and the forward lunge (b).** VPT = vibration perception threshold in volts, MM = medial malleolus. The box includes the first to the third quartile, and the median value is indicated by the black line through the box. The circles indicate outliers and the asterisks indicate extreme values. Stair descend, n = 22; forward lunge, n = 19.

Women with a KMFP during stair descending had significantly worse vibration sense at MTP1 and MM than those with a KOFP (mean difference, − 2.43 and 95% CI, −4.84 to −0.02 for MTP1; mean difference, −2.98 and 95% CI, −4.91 to −0.84 for MM). Women with a KMFP during the forward lunge had significantly worse vibration sense at MM than those with a KOFP (mean difference, 2.69 and 95% CI, −4.63 to −0.74), but no such differences were found in men (Table 6 and Figure 3a-b).

**Table 6:** **Sensory function in subjects with good and poor movement quality during the functional tasks**

	Functional tasks											
Measures of sensory function												
	Single-limb mini squat			Stair descending			Forward lunge			Drop-jump		
	(n = 7)	(n = 16)		(n = 13)	(n = 9)		(n = 8)	(n = 11)		(n = 7)	(n = 6)	
***Women***	Good	Poor	Mean difference (95%CI)	Good	Poor	Mean difference (95%CI)	Good	Poor	Mean difference (95%CI)	Good	Poor	Mean difference (95%CI)
	Mean (SD)	Mean (SD)		Mean (SD)	Mean (SD)		Mean (SD)	Mean (SD)		Mean (SD)	Mean (SD)	
**TDPM**	1.82 (0.81)	2.70 (1.52)	−0.88 (−2.16 to 0.39)	2.71 (1.54)	2.00 (1.17)	0.71 (−0.56 to 1.98)	2.59 (1.63)	2.55 (1.41)	0.48 (−1.43 to 1.53)	1.96 (1.25)	2.92 (1.77)	−0.95 (−2.80 to 0.89)
**VPT MTP1**	4.29 (1.11)	5.37 (2.67)	−1.01 (−3.28 to 1.11)	4.05 (0.99)	6.48 (3.10)	**−2.43 (−4.84 to −0.02)**	4.04 (1.16)	5.15 (2.28)	−1.10 (−2.97 to 0.75)	4.76 (1.47)	3.72 (0.57)	1.04 (−0.37 to 2.45)
**VPT MM**	6.52 (1.86)	7.42 (2.83)	−0.90 (−3.39 to 1.55)	5.97 (1.77)	8.95 (2.82)	**−2.88 (−4.91 to −0.84)**	5.25 (1.22)	7.94 (2.37)	**−2.69 (−4.63 to −0.74)**	6.52 (2.12)	6.17 (0.78)	0.36 (−1.67 to 2.38)
**VPT MF**	12.29 (5.66)	12.12 (2.61)	0.16 (−3.37 to 3.69)	11.43 (4.13)	13.59 (2.61)	−2.15 (−5.41 to 0.10)	10.33 (4.17)	13.67 (3.31)	−3.33 (−6.95 to 0.28)	10.62 (4.86)	13.50 (3.06)	−2.88 (−7.95 to 2.19)
***Men***	(n = 7)	(n = 21)		(n = 19)	(n = 9)		(n = 12)	(n = 16)		(n = 11)	(n = 12)	
**TDPM**	1.86 (0.86)	1.93 (0.89)	−0.07 (−0.86 to 0.72)	1.66 (0.58)	2.44 (1.14)	−0.79 (−1.69 to 0.11)	1.98 (1.03)	1.86 (0.75)	0.12 (−0.57 to 0.81)	1.57 (0.71)	2.23 (1.04)	−0.66 (−1.44 to 0.12)
**VPT MTP1**	5.24 (1.82)	5.29 (3.20)	−0.47 (−2.68 to 2.59)	5.58 (3.35)	4.63 (1.46)	0.95 (−1.46 to 3.37)	6.00 (4.08)	4,73 (1.43)	1.27 (−0.98 to 3.52)	6.12 (4.26)	4.86 (1.33)	1.29 (−1.43 to 3.95)
**VPT MM**	7.81 (1.92)	9.09 (4.53)	−1.29 (−5.28 to 2.71)	9.00 (5.16)	8.29 (2.31)	0.71 (−3.02 to 4.43)	9.94 (6.33)	7.89 (1.91)	2.05 (−1.38 to 3.93)	9.94 (6.53)	8.36 (2.31)	1.58 (−2.59 to 5.75)
**VPT MF**	12.90	14.36	−1.46 (−5.12 to 2.20)	13.77 (4.32)	14.48 (3.62)	−0.71 (−4.13 to 2.71)	14.92 (4.66)	13.31 (3.63)	1.60 (−1.57 to 4.78)	14.06 (4.84)	14.36 (3.94)	−0.30 (−4.17 to 3.57)

TDPM = threshold to detection of passive motion, VPT = vibration perception threshold, MTP1 = metatarsophalangeal joint 1, MM = medial malleolus, MF = medial femoral condyle, bold text = statistically significant difference (p ≤ 0.05).

## Discussion

These results indicate that worse kinesthetic acuity might be associated with KMFP during the drop jump—a test resembling a sports activity—in men with ACL injury. Worse vibration sense at the foot might be associated with KMFP during functional tasks resembling activities of daily living in women, but no such associations were observed in men.

Worse kinesthesia was related to a KMFP during the drop jump in men (r_s_ = 0.423) indicating that proprioceptive acuity contributes to movement quality at the knee during movements resembling sport-related activities. A similar, but non-significant, correlation coefficient was found in women (r_s_ = 0.469, p = 0.106). To reach a significant correlation of r_s_ = 0.47 would have required at least 18 subjects in the analysis [36], but we had only 13 women in the present study. Thus, the non-significant correlation in women might be due to too small of a sample. Moreover, in absolute values, although not reaching statistical significance, both women and men with KMFP during the drop jump had approximately 40% and 50% worse kinesthesia, respectively, than those with KOFP. This finding justifies further studies on the possible importance of proprioceptive acuity for medio-lateral knee position. Our hypothesis that the association between kinesthesia and movement quality would be more evident in women than in men was not confirmed because similar associations were observed for both genders. Because this is the first study on the association between sensory function and medio-lateral knee position during functional tasks, further studies are needed to confirm our findings.

Proprioceptive mechanoreceptors like the Golgi organ, free nerve endings, Pacinian corpuscles, and Ruffini endings are situated in the human ACL, capsule, menisci, and cartilage and are suggested to play an important role in the ability to detect movements and to sense where the joints are in relation to each other and to the environment [37, 38]. These receptors are also reported to be involved in muscle stiffness regulation via the gamma muscle spindle circuit and are thus also important for dynamic joint stability [38]. Consequently, damage to the ACL might lead to deterioration in these afferent sensory signals. The results from the present study indicate that ACL-injured individuals with worse proprioceptive acuity might have reduced ability to control medio-lateral knee joint motion during the drop jump. Future studies will reveal if this is true also for individuals without knee injury but who are at high risk of sustaining a knee injury such as high-level athletes.

There has recently been an increased interest in measuring vibration sense in patients with knee injury and knee or hip osteoarthritis [18, 28, 32]. These studies show that patients with hip or knee osteoarthritis have an impaired sense of vibration both in the affected and adjacent joints [28, 32]. However, no such impairments have yet been observed in subjects with meniscal or ACL injuries [18]. The possible associations between worse vibration sense and KMFP during stair descending and forward lunge in women in the present study could indicate some role of vibration sense for movement quality. No such relations were found in men, however, and the underlying reason for these gender differences needs further study along with the possible relevance of vibratory deficiencies in these patients.

In contrast to a previous study that reported that women with ACL injury were more prone to a KMFP compared to their male counterparts during a single leg squat [5], we found no gender-difference in medio-lateral knee position during the different functional tasks, including the single leg squat. There are several differences between that study and our study regarding outcomes and participants that might explain the contrasting findings. We assessed KMFP by visual observation in high-level athletes 9–16 months after injury, and all of our subjects were participating in a thorough neuromuscular training program at a sport rehabilitation clinic under the supervision of experienced physical therapists. In the previous study, Yamazaki et al. [5] used 3-D equipment to assess knee joint movement in recreational athletes at 3–3.5 months after injury without any rehabilitation training.

The underlying mechanisms for a KMFP in patients with ACL injury are still unclear. Trulsson et al. reported that a KMFP was more often present in patients with ACL injury than in healthy controls during different functional activities such as the forward lunge and a mini squat [4]. In another study, a higher degree of 3-D knee abduction was found in the subjects’ ACL-injured knee compared to the non-injured knee during a single leg squat [5]. However, possible contributing factors for the medio-lateral knee position during functional tasks are poorly investigated in patients with ACL injury. The correlations found in the present study were mostly moderate and indicated some association between sensory function and medio-lateral knee position. However, future studies are warranted to evaluate the role of sensory function as well as other possible contributing sensorimotor and biomechanical factors for medio-lateral knee position in patients with knee injury. Such knowledge will help in the design of training programs for these patients.

There are some limitations in this study. Sixteen subjects were not able to perform all functional tasks, for example, 10 out of the 23 women were not able to execute the drop jump task. This resulted in a small sample for the drop jump in women and a small number of subjects in the good and poor knee-position groups during all functional tasks, and this might have contributed to the lack of significant findings. This study had a cross-sectional design and we are, therefore, unable to draw any conclusions regarding causal relationships. Moreover, all subjects were athletes competing at a high level before they were injured, primarily soccer or team handball, and our results might not be generalizable to a recreational or sedentary population. Finally, because this was an exploratory study we did not correct for multiple comparisons. Thus there is a risk that some of the findings in this study are due to chance, and further studies are needed to confirm our results.

## Conclusion

Our findings suggest that impaired proprioceptive acuity, measured as TDPM, might be associated with a medial position of the knee relative to the foot during the drop jump in men with ACL injury. Decreased vibration sense at the foot and the knee might be associated with worse KMFP in women. However, further studies are needed to confirm these results and to determine the relative contribution of proprioception, vibration sense, and other aspects of sensorimotor function on KMFP.

## Authors’ original submitted files for images

Below are the links to the authors’ original submitted files for images. Authors’ original file for figure 1 Authors’ original file for figure 2 Authors’ original file for figure 3 Authors’ original file for figure 4 Authors’ original file for figure 5 Authors’ original file for figure 6 Authors’ original file for figure 7 Authors’ original file for figure 8
