# Supplementary figures and images for: Association between sensory function and medio-lateral knee position during functional tasks in patients with anterior cruciate ligament injury
Source: BMC Musculoskelet Disord. 2014 Dec 13;15:430. doi: 10.1186/1471-2474-15-430 (PMC4301659; doi:10.1186/1471-2474-15-430)

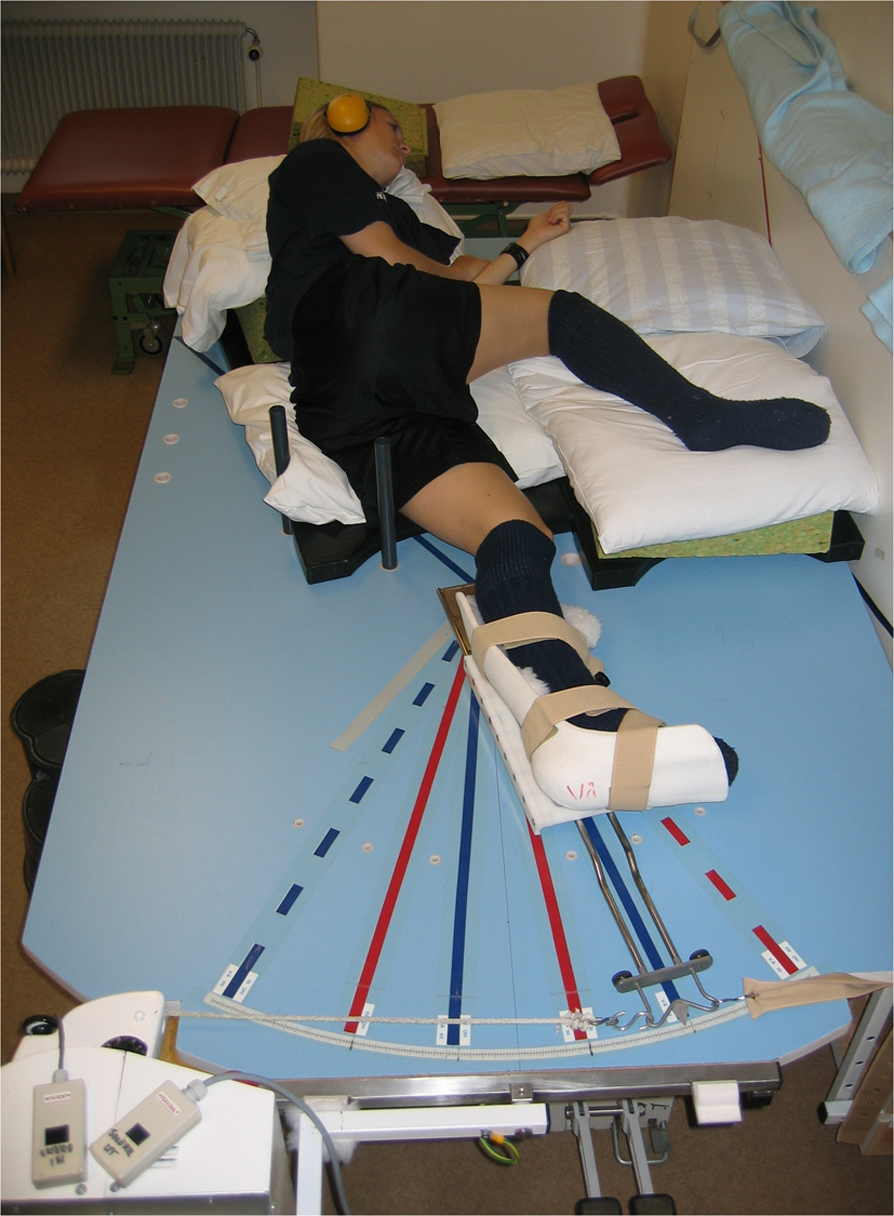

Supplement: Supplementary file 1 — Authors’ original file for figure 1 [file 12891_2014_2356_MOESM1_ESM.tif]

a

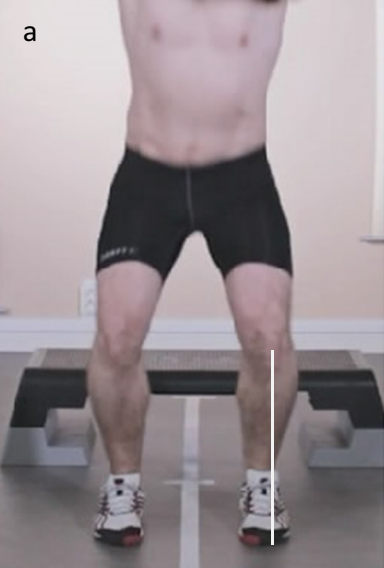

b

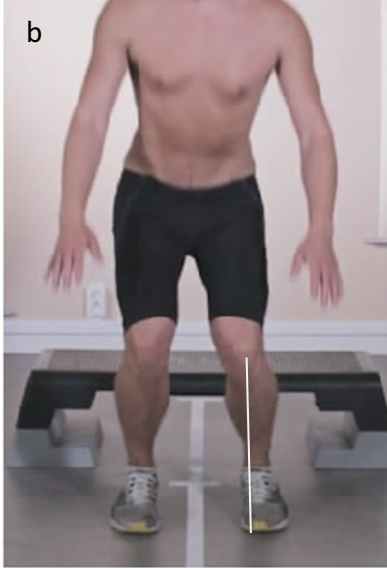

c

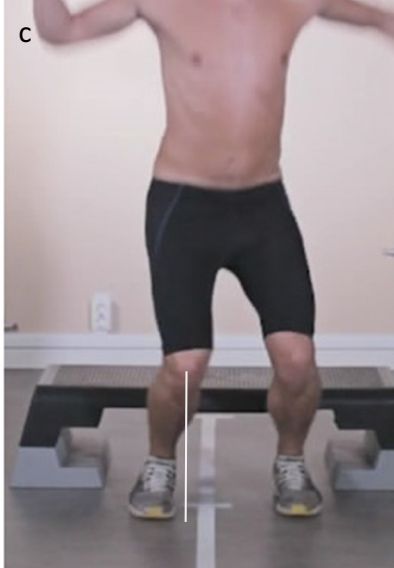

Supplement: Supplementary file 2 — Authors’ original file for figure 2 [file 12891_2014_2356_MOESM2_ESM.pdf]

a

VPT - MM (Volt)

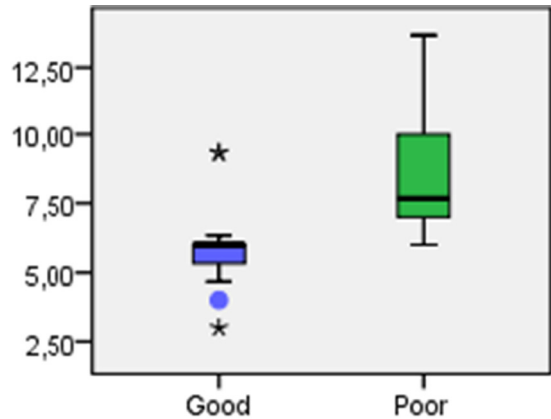

Stair descend - Women

b

VPT - MM (Volt)

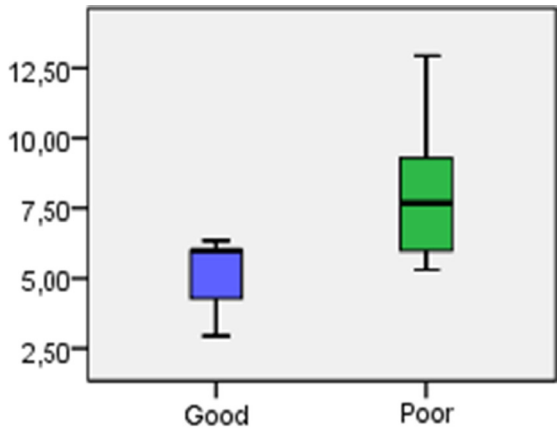

Forward lunge - Women

Supplement: Supplementary file 3 — Authors’ original file for figure 3 [file 12891_2014_2356_MOESM3_ESM.pdf]

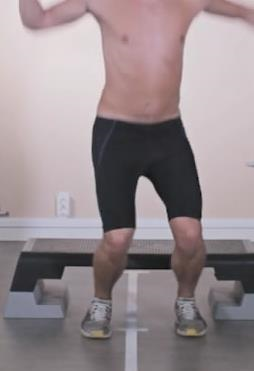

Supplement: Supplementary file 4 — Authors’ original file for figure 4 [file 12891_2014_2356_MOESM4_ESM.png]

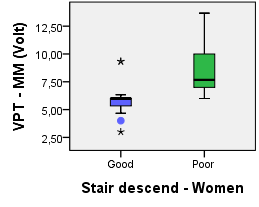

Supplement: Supplementary file 5 — Authors’ original file for figure 5 [file 12891_2014_2356_MOESM5_ESM.tiff]

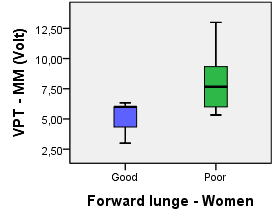

Supplement: Supplementary file 6 — Authors’ original file for figure 6 [file 12891_2014_2356_MOESM6_ESM.tiff]

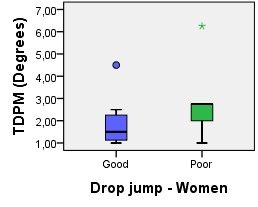

Supplement: Supplementary file 7 — Authors’ original file for figure 7 [file 12891_2014_2356_MOESM7_ESM.tiff]

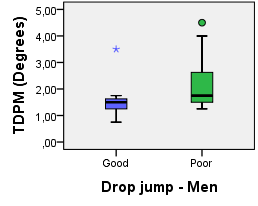

Supplement: Supplementary file 8 — Authors’ original file for figure 8 [file 12891_2014_2356_MOESM8_ESM.tiff]
